# NmcA Carbapenem-hydrolyzing Enzyme in *Enterobacter cloacae* in North America^1^

**DOI:** 10.3201/eid0908.030096

**Published:** 2003-08

**Authors:** Sudha Pottumarthy, Ellen Smith Moland, Stefan Juretschko, Susan R. Swanzy, Kenneth S. Thomson, Thomas R. Fritsche

**Affiliations:** * University of Washington School of Medicine, Seattle, Washington, USA; † Creighton University Medical Center, Omaha, Nebraska, USA

**Keywords:** Antibiotic resistance, beta-lactamase, carbapenem, *Enterobacter cloacae*, *dispatch*

## Abstract

An imipenem-resistant *Enterobacter cloacae* isolate was recovered from the blood of a patient with a hematologic malignancy. Analytical isoelectric focusing, inhibitor studies, hydrolysis, induction assays, and molecular sequencing methods confirmed the presence of a NmcA carbapenem-hydrolyzing enzyme. This first report of NmcA detected in North America warrants further investigation into its distribution and clinical impact.

Carbapenem resistance mediated by acquired carbapenemases is a growing concern worldwide (*1*–*3*). Carbapenems are the most potent of β-lactam antibiotics with a broad spectrum of activity against aerobic and anaerobic bacterial pathogens. These antibiotics are also the most reliable of the β-lactams for treatment of infections caused by organisms that produce extended-spectrum β-lactamases (ESBLs) or AmpC β-lactamases (*4*,*5*). Intrinsic resistance does occur, however, in a number of species, including *Bacillus cereus*, *Stenotrophomonas maltophilia*, *Flavobacterium* sp. and *Chryseobacterium* sp., *Aeromonas hydrophila*, *Legionella gormanii,* and *Janthinobacterium lividum,* because of the presence of metallo-β-lactamases (*1*). Acquired carbapenemases, on the other hand, are a heterogeneous mixture of enzymes that belong to Ambler class A, B, or D enzymes and may be plasmid or chromosomally encoded (*1*,*2*). Here we describe the characterization of imipenem resistance attributable to an acquired class A carbapenem-hydrolyzing enzyme, NmcA, in an *Enterobacter cloacae* isolate.

## The Study

A 33-year-old man with acute myeloid leukemia was admitted to a hospital in Seattle, Washington, with neutropenic fever; he was initially treated with ceftazidime. His therapy was changed to acyclovir and imipenem because of unremitting fevers and oral lesions positive for herpes simplex virus type 1, whereupon his fever defervesced immediately. The fever recurred 2 weeks after treatment was begun, and imipenem-resistant *E. cloacae* was isolated from the blood. After the results of susceptibility testing were obtained, his therapy was changed to levofloxacin, and he exhibited a good clinical response.

**Figure 1:**
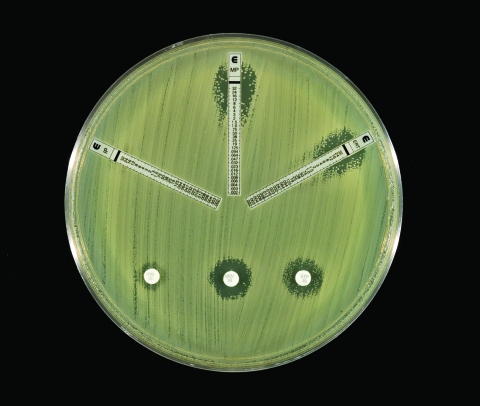
Susceptibility testing of *Enterobacter cloacae* to carbapenems. Both methods had ill-defined zones of inhibition with inner colonies growing up to the disks or the E-test strips, respectively.

**Figure 2:**
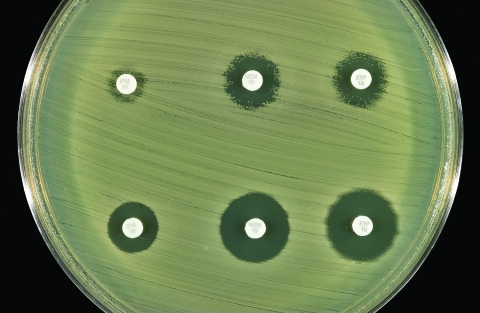
Effect of addition of clavulanic acid (10 μL of 1,000 μg/mL) to the zones of inhibition of the three carbapenem disks. Top row (left to right): imipenem, meropenem, and ertapenem disks without clavulanic acid. Bottom row (left to right): imipenem, meropenem, and ertapenem disks with clavulanic acid.

The *E. cloacae* isolate was susceptible to piperacillin, piperacillin-tazobactam, ceftazidime, ceftriaxone, cefepime, ciprofloxacin, gentamicin, aztreonam, and trimethoprim-sulfamethoxazole and was resistant to ampicillin, amoxicillin-clavulanic acid, cefazolin, and cefoxitin. By both disk diffusion and E-test methods (AB Biodisk, Solna, Sweden), the zones of inhibition around the imipenem, meropenem, and ertapenem disks were indistinct, with inner colonies extending up to the disk or the highest concentration on the E-test strip (MIC >32 μg/mL) and were interpreted as resistant (Figure 1). Addition of 10 μL of 1,000 μg/mL clavulanic acid to the imipenem, meropenem, and ertapenem disks resulted in clearly defined and enlarged zones of inhibition and loss of the inner colonies (Figure 2). Using either cefoxitin or imipenem as inducers by the disk induction method resulted in the formation of blunted or D-shaped zones of inhibition to meropenem and ertapenem (*6*). We performed a carbapenem bioassay by incubating 100 μL of a 50 μg/mL solution of each carbapenem (imipenem, meropenem, or ertapenem) with 100 μL of either crude β-lactamase extract or phosphate-buffered saline for 90 min at room temperature. The inactivating capacity of the enzyme was then tested by a disk diffusion assay that demonstrated hydrolysis of imipenem, meropenem, and ertapenem (*7*).

**Figure 3:**
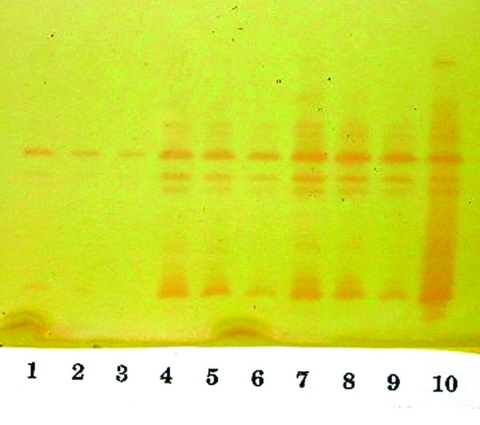
Analytical isoelectric focusing (IEF) of serially diluted broth induced enzymes. The isoelectric points (pIs) of the two prominent bands midway and at the bottom of the gel are 6.9 and >9.0. Lanes 1–3: uninduced enzyme serially diluted 1:1, 1:2, and 1:4. Lanes 4–6: enzyme induced with 8 μg/mL of cefoxitin serially diluted 1:1, 1:2, and 1:4. Lanes 7–9: enzyme induced with 4 μg/mL of imipenem serially diluted 1:1, 1:2 and 1:4. Lane 10: NOR-1 control, induced with 16 μg/mL of cefoxitin.

Isoelectric focusing (IEF) to determine isoelectric points (pIs) and general inhibitor characteristics with an ampholine polyacrylamide gel (pH range 3.5–9.5) on a flatbed apparatus (Multiphor LKB, Bromma, Stockholm, Sweden) indicated that this organism produced a β-lactamase enzyme with a pI of 6.9 that was inhibited by clavulanic acid and not by cloxacillin (*8*,*9*). This enzyme hydrolyzed 1 μg/mL imipenem on the IEF gel overlay. Inducibility of the enzyme by imipenem (4 mg/L) and cefoxitin (8 mg/L) was confirmed by the broth induction method and IEF (*10*). After the broth induction procedure, the uninduced and induced preparations were serially diluted in 0.1 M potassium phosphate buffer, pH 7.0, permitting both qualitative and semiquantitative assessment of enzyme activity among the various β-lactamases in the isolate, as determined by IEF. The broth induction enzyme preparation from a 100-mL, 3.5-h Mueller-Hinton broth produced a visible but light AmpC band, pI >9.0. The induction with cefoxitin and imipenem increased the levels of both the AmpC and the pI 6.9 carbapenem-hydrolyzing enzyme, as visualized in Figure 3. A cefoxitin-induced preparation of *E. cloacae* NOR-1 (that produces NmcA) was included as a control strain for IEF (*11*).

Chromosomal DNA was extracted from overnight cultures with the Qiagen DNeasy Tissue kit (QIAGEN Inc., Valencia, CA). Polymerase chain reaction (PCR)-amplification with a thermal cycler (MJ Research PTC - 200-DNA Engine, San Francisco, CA) was used to obtain a 2,122 base pair gene fragment consisting of the carbapenemase structural gene *nmcA* and its regulatory gene *nmcR* (*12*,*13*). The nucleotide sequences of the primers were 5′- TGC CAG CTT AAT TAT TTT CAG ATT AG -3′ (*nmcR* positions 32–56) and 5′- ATT TTT TTC ATG ATG AAG TTA AGC C -3′ (*nmcA* positions 2,129–2,154). PCR amplification of the *nmcR/nmcA* genes showed positive results with both NOR-1 and the strain under study, suggesting that the latter strain harbored a carbapenem-hydrolyzing enzyme similar to that of NOR-1 (*11*). Subsequent sequence analysis of the amplified DNA-region showed a similarity of 100% (*2*,122 base pairs) between the sequence of our strain and the carbapenemase genes, regulatory (*nmcR*) and structural (*nmcA*) genes of the *E. cloacae* strain NOR-1 (*13*).

## Conclusions

Ambler class A carbapenem-hydrolyzing enzymes are relatively rare and have been reported to date in only a handful of isolates of *Enterobacteriaceae* worldwide (*1*,*2*,*14*–*16*). Sme-1 was identified from two *Serratia marcescens* strains collected in London in 1982, and IMI-1 was found in two strains of *E. cloacae* isolated in southern California in 1984. Both enzymes were retrospectively characterized from clinical isolates recovered before carbapenems were marketed (*17*,*18*). Subsequently, imipenem-resistant *S. marcescens* strains were identified from Minnesota, California, and Boston during a 15-year period, 1985–1999. The Minnesota isolate produced an enzyme identical to the London Sme-1 enzyme, but the Boston and California strains produced a related Sme-1 enzyme with a single amino-acid variation, named Sme-2. More recently, a fourth class A β-lactamase, KPC-1, has been described in a strain of *Klebsiella pneumoniae* collected in North Carolina (*15*).

NmcA was the first class A carbapenemase identified in a clinical isolate of *E. cloacae*, NOR-1, in 1990, following the introduction of carbapenems (*11*). This strain was recovered from the pus of a fistulized subcutaneous abscess of a hospitalized French patient who had received one intravenous bolus (500 mg) of imipenem before the strain isolation. The present strain was also isolated from the patient during therapy with imipenem. Similar to the NOR-1 isolate, this strain displayed decreased susceptibility to carbapenems (imipenem and meropenem) while remaining fully susceptible to extended-spectrum cephalosporins (*1*). The strain also had decreased susceptibility to ertapenem. Although the zones of inhibition around the carbapenem disks were poorly defined, leading us to question the purity of the isolate, repeated testing confirmed our initial observations. The carbapenem-hydrolyzing enzyme was inhibited by clavulanic acid, as shown by the disk diffusion method and confirmed by the IEF overlay method, similar to the other class A carbapenem-hydrolyzing enzymes (*1*,*2*). We found that adding of clavulanic acid to the carbapenem disks, similar to the method used for National Committee for Clinical Laboratory Standards ESBL confirmatory testing, was a simple and helpful diagnostic approach. Because other investigators have defined the hydrolytic spectrum of the NmcA enzyme, we have not established whether the NmcA enzyme was solely responsible for the carbapenem resistance in our isolate (*11*,*13*).

Class A carbapenem-hydrolyzing β-lactamases are usually coproduced with other β-lactamases, conferring distinct biochemical advantages to the parent strain (*3*). The carbapenem-hydrolyzing enzyme IMI-1 was produced along with AmpC and TEM-type enzymes; Sme-1 was co-produced along with a chromosomal AmpC (*3*,*18*). Our strain was similar to NOR-1 and produced an AmpC enzyme of pI >9.0 along with the carbapenem-hydrolyzing enzyme of pI 6.9. The inducibility of NmcA in this strain of *E. cloacae* by cefoxitin and imipenem was evident by disk induction and confirmed by broth induction with IEF, similar to the NOR-1 strain. Expression of NmcA has been shown to be inducible because of the presence of a LysR-type regulatory gene, Nmc-R, which precedes the bla_NMC-A_ gene (*1*). NmcR mediates biosynthesis of the carbapenemase enzyme at the basal state, which is further increased when β-lactam-mediated induction occurs (*13*). Naas et al. have shown that NmcR acts as a positive regulator for carbapenemase biosynthesis even in the absence of an inducer (unlike Amp-R, which is a negative regulator of the cephalosporinase expression in the AmpC-AmpR system) and deletion of NmcR results in a sharp decrease in carbapenem MIC and loss of β-lactamase inducibility (*13*). A recent study has shown that AmpD from *E. cloacae* NOR-1 is involved in the regulation of expression of both β-lactamases (NmcA and AmpC) (*19*). These findings suggest that different structural genes may be under the control of identical regulatory systems and that nucleotide substitutions in AmpD could lead to the stable co-expression of the NmcA together with overexpression of the AmpC enzyme in *E. cloacae* (*19*).

This is the first report of the isolation of the NmcA carbapenem-hydrolyzing enzyme from a clinical isolate of *E. cloacae* in North America. The importance of this finding for clinicians and laboratorians is illustrated by the following: the NmcA enzyme is able to hydrolyze carbapenems; the NmcA enzyme occurred in *E. cloacae* (a common nosocomial pathogen); the NmcA enzyme was difficult to detect; the NmcA enzyme is inducible; and the expression of NmcA is coregulated with AmpC. Accurately identifying the different mechanisms of carbapenem resistance will help determine the epidemiology, risk factors, and therapeutic options in each case. The continued emergence of novel resistance mechanisms to carbapenems worldwide reemphasizes the need not only for prudent carbapenem use but also for prudent antibiotic use in general.

## References

[1] Nordmann P, Poirel L. Emerging carbapenemases in Gram-negative aerobes. Clin Microbiol Infect. 2002;8:321–31. 10.1046/j.1469-0691.2002.00401.x 12084099

[2] Livermore DM, Woodford N. Carbapenemases: a problem in waiting? Curr Opin Microbiol. 2000;3:489–95. 10.1016/S1369-5274(00)00128-4 11050448

[3] Rasmussen BA, Bush K. Carbapenem-hydrolyzing beta-lactamases. Antimicrob Agents Chemother. 1997;41:223–32. 9021171 10.1128/aac.41.2.223 PMC163693

[4] Bradford PA. Extended-spectrum beta-lactamases in the 21st century: characterization, epidemiology, and detection of this important resistance threat. Clin Microbiol Rev. 2001;14:933–51. 10.1128/CMR.14.4.933-951.2001 11585791 PMC89009

[5] Philippon A, Arlet G, Jacoby GA. Plasmid-determined AmpC-type beta-lactamases. Antimicrob Agents Chemother. 2002;46:1–11. 10.1128/AAC.46.1.1-11.2002 11751104 PMC126993

[6] Sanders CC, Sanders WE Jr. Emergence of resistance during therapy with the newer beta-lactam antibiotics: role of inducible beta-lactamases and implications for the future. Rev Infect Dis. 1983;5:639–48. 6353526 10.1093/clinids/5.4.639

[7] Masuda G, Tomioka S, Hasegawa M. Detection of beta-lactamase production by gram-negative bacteria. J Antibiot. 1976;29:662–4. 950322 10.7164/antibiotics.29.662

[8] Thomson KS, Sanders CC, Washington JA II. High level resistance to cefotaxime and ceaftazidime in *Klebsiella pneumoniae* isolates from Cleveland, Ohio. Antimicrob Agents Chemother. 1991;35:1001–3. 1854155 10.1128/aac.35.5.1001 PMC245146

[9] Sanders CC, Sanders WE Jr, Moland ES. Characterization of β-lactamases in situ on polyacrylamide gels. Antimicrob Agents Chemother. 1986;30:951–2. 3492960 10.1128/aac.30.6.951 PMC180628

[10] Pitout JD, Thomson KS, Hanson ND, Ehrhardt AF, Coudron P, Sanders CC. Plasmid-mediated resistance to expanded-spectrum cephalosporins among *Enterobacter aerogenes* strains. Antimicrob Agents Chemother. 1998;42:596–600. 9517938 10.1128/aac.42.3.596 PMC105504

[11] Nordmann P, Mariotte S, Naas T, Labia R, Nicolas MH. Biochemical properties of a carbapenem-hydrolyzing beta-lactamase from *Enterobacter cloacae* and cloning of the gene into *Escherichia coli.* Antimicrob Agents Chemother. 1993;37:939–46. 8517720 10.1128/aac.37.5.939 PMC187856

[12] Sanger F, Nicklen S, Coulson AR. DNA sequencing with chain-terminating inhibitors. Proc Natl Acad Sci U S A. 1977;74:5463–7. 10.1073/pnas.74.12.5463 271968 PMC431765

[13] Naas T, Nordmann P. Analysis of a carbapenem-hydrolyzing class A beta-lactamase from *Enterobacter cloacae* and of its LysR-type regulatory protein. Proc Natl Acad Sci U S A. 1994;91:7693–7. 10.1073/pnas.91.16.7693 8052644 PMC44468

[14] Queenan AM, Torres-Viera C, Gold HS, Carmeli Y, Eliopoulos GM, Moellering RC Jr, SME-type carbapenem-hydrolyzing class A beta-lactamases from geographically diverse *Serratia marcescens* strains. Antimicrob Agents Chemother. 2000;44:3035–9. 10.1128/AAC.44.11.3035-3039.2000 11036019 PMC101599

[15] Yigit H, Queenan AM, Anderson GJ, Domenech-Sanchez A, Biddle JW, Steward CD, Novel carbapenem-hydrolyzing beta-lactamase, KPC-1, from a carbapenem-resistant strain of *Klebsiella pnuemoniae.* Antimicrob Agents Chemother. 2001;45:1151–61. 10.1128/AAC.45.4.1151-1161.2001 11257029 PMC90438

[16] Moland ES, Hanson ND, Herrera VL, Black JA, Lockhart TJ, Hossain A, Plasmid-mediated carbapenem-hydrolyzing β-lactamase, KPC-2, in *Klebsiella pneumoniae* isolates.. J Antimicrob Chemother. 2003;51:711–4. 10.1093/jac/dkg124 12615876

[17] Yang YJ, Wu PJ, Livermore DM. Biochemical characterization of a beta-lactamase that hydrolyzes penems and carbapenems from two *Serratia marcescens* isolates. Antimicrob Agents Chemother. 1990;34:755–8. 2193618 10.1128/aac.34.5.755 PMC171686

[18] Rasmussen BA, Bush K, Keeney D, Yang Y, Hare R, O’Gara C, Characterization of IMI-1 beta-lactamase, a class A carbapenem-hydrolyzing enzyme from *Enterobacter cloacae.* Antimicrob Agents Chemother. 1996;40:2080–6. 8878585 10.1128/aac.40.9.2080 PMC163477

[19] Naas T, Massuard S, Garnier F, Nordmann P. AmpD is required for regulation of expression of NmcA, a carbapenem-hydrolyzing beta-lactamase of *Enterobacter cloacae.* Antimicrob Agents Chemother. 2001;45:2908–15. 10.1128/AAC.45.10.2908-2915.2001 11557489 PMC90751

